# Killing Two Birds with One Stone: Natural Rice Rhizospheric Microbes Reduce Arsenic Uptake and Blast Infections in Rice

**DOI:** 10.3389/fpls.2016.01514

**Published:** 2016-10-13

**Authors:** Venkatachalam Lakshmanan, Jonathon Cottone, Harsh P. Bais

**Affiliations:** ^1^Department of Plant and Soil Sciences, University of DelawareNewark, DE, USA; ^2^Delaware Biotechnology InstituteNewark, DE, USA

**Keywords:** aboveground, arsenic, belowground, microbiome, *M. oryzae*, rhizosphere

## Abstract

Our recent work has shown that a rice thizospheric natural isolate, a *Pantoea sp* (hereafter EA106) attenuates Arsenic (As) uptake in rice. In parallel, yet another natural rice rhizospheric isolate, a *Pseudomonas chlororaphis* (hereafter EA105), was shown to inhibit rice blast pathogen *Magnaporthe oryzae*. Considering the above, we envisaged to evaluate the importance of mixed stress regime in rice plants subjected to both As toxicity and blast infections. Plants subjected to As regime showed increased susceptibility to blast infections compared to As-untreated plants. Rice blast pathogen *M. oryzae* showed significant resistance against As toxicity compared to other non-host fungal pathogens. Interestingly, plants treated with EA106 showed reduced susceptibility against blast infections in plants pre-treated with As. This data also corresponded with lower As uptake in plants primed with EA106. In addition, we also evaluated the expression of defense related genes in host plants subjected to As treatment. The data showed that plants primed with EA106 upregulated defense-related genes with or without As treatment. The data shows the first evidence of how rice plants cope with mixed stress regimes. Our work highlights the importance of natural association of plant microbiome which determines the efficacy of benign microbes to promote the development of beneficial traits in plants.

## Introduction

Rice is the food for over half the world's population and contributes as much as 80% of the daily caloric intake in many South Asian countries (Dawe et al., 2010). Tragically, paddy rice grown in South East Asia and particularly in the arsenic (As) hotspots accumulates inorganic As leading to elevated arsenic in rice grain, contributing to large-scale mass As-poisoning (Huq, 2008). Severe As intoxication results in skin lesions and neurological injury. Chronic low-level exposure increases incidence of multiple cancers and causes disfigurement and recurring diarrhea. This is exacerbated by the fact that elevated As concentrations in soil is phytotoxic and can contribute to decreased grain fill, lowered yield and reduced food availability (Abedin et al., 2002; Panaullah et al., 2009). Novel strategies are needed to simultaneously decrease the As content and increase nutritional value in rice grains.

The effects of beneficial rhizospheric microbes on soil structure and chemistry are well known (Bais et al., 2006), but little is known about the physiology and biochemistry underlying the interactions between bacteria and elemental cycling in soil that influence plant yield and productivity. Plant-associated bacteria within the rhizosphere have the capability to modulate the uptake of elements, including Fe and As. Rhizospheric microbes can alter the rhizosphere geochemistry, potentially rendering As less soluble by tying it up with Fe oxides (Zhu et al., 2013). We have identified a suite of nonpathogenic, rice-associated bacteria, *Pantoea sp.* (EA106) from roots of rice grown in North American rice paddy fields that have shown they promote healthy rice growth and enhance the oxidizing potential of the rhizosphere (Lakshmanan et al., 2015). This creates a microenvironment where As is conceptually less available for plant assimilation in the immediate vicinity of the root by being tightly bound to Fe oxides that form on or near the plant roots, so-called Fe-plaque. It is shown that application of EA106 increases Fe mobilization to roots leading to formation of Fe-plaques and reduced As uptake to shoots (Lakshmanan et al., 2015).

Rice is the principal food crop for more than half of the world's population, but yields are reduced significantly due to disease pressure. Each year rice blast disease caused by *Magnaporthe oryzae* destroys enough rice to feed an estimated 60 million people (Zeigler et al., 1994). In Asia, rice is grown widely under rain-fed, lowland conditions and approximately 45% of the total rice area is not irrigated (Manickavelu et al., 2006; Dawe et al., 2010). Therefore, crops grown in this condition are subjected to both abiotic and biotic stresses, and data show that rice is more susceptible to *M. oryzae* during drought stress (Mosquera et al., 2009). Significant progress has been made to generate plant lines with improved resistance, but the pathogen rapidly overcomes plant-encoded resistance. Chemical pesticides also offer marginal protection from the disease, and there are limited biocontrol strategies against any foliar pathogens of rice. Previously, we have discovered a microbe from rice rhizosphere, *Pseudomonas chlororaphis* (EA105) which attenuates *M. oryzae* in *vitro* and *in vivo* (Spence et al., 2014a,b, 2015). The microbes naturally associate with healthy rice roots and are readily cultured and introduced to axenically-grown rice or rice previously infected with other microbes.

Plants are often confronted with both biotic and abiotic stress leading to loss of productivity and yield (Ben Rejeb et al., 2014). It is often argued that combination of both abiotic and biotic stress responses in plants may be beneficial to plant performance against biotic stress responses (AbuQamar et al., 2009; Ben Rejeb et al., 2014). Interestingly, plants exposed to bacterial pathogens lead to stress responses while dealing with one specific stress is often referred to as “cross tolerance” (Suzuki et al., 2012). Conventionally, plants usually compartmentalize while confronting more than one stress response at time. It is argued that plants are able to defend themselves facing one stress and simultaneously become more resistant to other stress regimes (Bowler and Fluhr, 2000). For example, it is shown that mechanical wounding in plants often results in increased resistance against abiotic stress such as salinity response (Capiati et al., 2006). Pre-exposure of plants against *Pseudomonas syringae* leads to insect tolerance in tomato against *Helicoverpa zea* (Stout et al., 1999). Along similar lines, exposure of plants against ozone increases resistance against *P. syringae* strains (Sharma et al., 1996; Borsani et al., 2001). The results were similar wherein plants were challenged with biotic stress first and then exposed to abiotic stress regime. It is shown that, plants exposed to aerial pathogens close stomata leading to drought tolerance (Goel et al., 2008). Interestingly, exposure of plants to benign microbiome also changed the aboveground physiology leading to resistance against the biotic stress (Reviewed by Lakshmanan et al., 2014; Pieterse et al., 2014). Conversely, plants interacting with simultaneous biotic and abiotic stress may lead to trade-offs increasing plant growth and fitness. The signaling pathways involved in plants exposed to multiple stresses are still being researched and how signaling pathways relate to traits for plant fitness and protection needs to be elucidated.

We have identified a suite of nonpathogenic, rice-associated bacteria (EA106 and EA105) from roots of rice grown in rice paddy fields and have shown that they promote healthy rice growth and enhance the oxidizing potential of the rhizosphere and induce plant protection against rice blast fungus (Spence et al., 2014a,b, 2015; Lakshmanan et al., 2015). The incidence of evaluating two different stress regimes in a model plant system is new. We have examined the fitness of rice plants exposed to As and *M. oryzae* infections simultaneously. The goal of our study was to see if plants exposed to As show variation in its response against *M. oryzae* infections. The impact of benign microbes under multiple stress regimes for defense-related genes and As uptake was also evaluated.

## Materials and methods

### Plant growth conditions

Rice cultivar Nipponbare seeds were provided by Genetic Stocks—Oryza (GSOR) Collection Dale Bumpers National Rice Research Center, Stuttgart, Arkansas, USA. Hyper-susceptible genotype Seraceltik (a gift from the Donofrio lab; University of Delaware, DE) was used for *M. oryzae* infections. Seeds were de-husked, surface sterilized with 50% commercial laundry bleach for 5 min and rinsed three times with sterile water. The surface sterilized seeds were transferred on a germination paper disk (single paper disk per plate and moist with 5 ml of sterile water) in a Petri dish and incubated on culture rack at room temperature (25 ± 2°C) with a photoperiod of 12 h dark and 12 h light (130 ± 20 μmol m^−2^s^−1^) for 7 days. Uniform-size seedlings were selected and used for hydroponic cultivation and *in vitro* experiments

### Preparation of bacterial EA106 and EA105

Bacterial strains EA105, a *Pseudomonas chlororaphis* (Spence et al., 2014a), and EA106, a *Pantoea sp* (Lakshmanan et al., 2015) were isolated from rice cultivar M-104 grown in the paddy soil by Dr. Venkatesan Sundaresan's lab, University of California (Davis). Freezer-stored glycerol stocks of EA106 and EA105 were streaked onto low-salt Luria-Bertani (LB) plates (10 g L^−1^ Tryptone, 5 g L^−1^ yeast extract, 5 g L^−1^ NaCl) and incubated at 30°C for overnight. An LB liquid culture was made with a single colony from plates and after 12 h incubation at 30°C with 180 rpm, when the OD_600_ reached 0.8–1.0, bacterial cells were centrifuged and washed in 10 mM MgCl_2_ to remove the medium and centrifuged and re-suspended in water to obtain desired inoculation density.

### Hydroponic cultivation of rice

Rice cultivar Seraceltik plants grown hydroponically as described by Lakshmanan et al. (2015). Briefly, 7-day-old, *in vitro*-germinated seedlings were transplanted into the hydroponic system that contained 8 L of rice nutrient solution. The hydroponic pots were incubated for 21 days in a growth chamber at 22 ± 2°C temperature, 14/10 h of light/dark photoperiod, 130 ± 20 μmol m^−2^s^−1^ light intensity and 80% relative humidity and the solution was renewed every 7 days. Then, plants were treated with 5 μM As(III), or EA105 (OD_600_ = 0.02) or EA106 (OD_600_ = 0.02) or combinations of all and grown for an additional 7 days, during which the pH was checked and adjusted daily (pH 6.0–6.5). After 7 days of treatments, samples were collected for *M. oryzae* infection and analyses for total As content.

### *M. oryzae* infection assay

Wildtype fungi *M. oryzae* 70-15, a fully sequenced, filter paper stocks stored at −20°C were inoculated onto oatmeal agar plates (OM: oatmeal 50 gL^−1^ and agar 15 gL^−1^) for germination and to establish starter cultures. Fungus grown on OM plates were transferred to complete medium (CM) containing sucrose (10 gL^−1^), cas-amino acids (6 gL^−1^), yeast extract (6 gL^−1^), and 1 mL of *Aspergillus nidulans* trace elements and kept in dark at 25°C for 7 days. The cultures were subsequently transferred to OM agar for 10 days for sporulation. Conidia from *M. oryzae* grown on OM agar plates were harvested, filtered through miracloth, and counted using a hemacytometer. Concentration of the conidial suspension was adjusted to 1 × 10^4^ spores mL^−1^. For *M. oryzae* infections, 7 days post treatment of Arsenic and rhizobacteria, hydroponically grown plant, the second youngest fully developed leaf was cut and affixed to a large 150 mm diameter petri dish, on top of moistened paper towels and treated with 3–20 μL droplets of spores were placed along the length on each leaf. Spore droplets were wiped after 24 h incubation in the dark at 25°C. After 120 h post treatment, length and width of lesions were measured also photographed.

### Exposure of as against *M. oryzae* and other non-host fungi

The non-host pathogenic fungi, *Fusarium equisetti* and *Geotrichum candidum* were acquired from Nancy Gregory at the University of Delaware and were grown on potato dextrose agar (PDA) for 7 days in dark at 25°C. Arsenic at different concentrations [(0–2000 μM As(III) or As(V)] were used to test against both *M. oryzae* and non-host fungi. Five mm plugs of fungal mycelia were placed in the center of the plates and plates were sealed with parafilm and put in the dark in a 25°C incubator. Photographs were taken after 5 days and the diameter of the mycelium growing out from the plug was measured. Percentage (%) inhibition was calculated by the formula:% inhibition = [(C–T) × 100)/C], where C = fungal diameter (cm) in the control plate, and T = fungal diameter (cm) in the As treated plates.

### Quantification of total as content

Plants grown in an 8-L hydroponic system for 21 days and treated with As(III), EA105 and EA106 as mentioned above were used for quantification of arsenic. After 7 days of treatments, the root, and shoot samples were collected and dried at 65°C for 3 days and weighed. The total concentrations As in the leaves and seeds were measured by ICP-OES at UD soil testing lab, University of Delaware.

### Spore germination and appresoria formation in *M. oryzae* treated with as

*M. oryzae* spores were grown on oatmeal agar and harvested and adjusted to concentration of 1 × 10^5^ spores mL^−1^. Then treated with mock, 5 μM, 10 μM, and 100 μM of As(III) and 50 μl were placed on the hydrophobic (Pho) surface of sterile GelBond. The GelBond films were placed in petri dishes with wet filter disks in the center to promote humidity. Plates were sealed and incubated in the dark for 12 h. Germination percentages were calculated after 6 h incubation, and appressoria formation was determined after 12 h. Appresoria formation was imaged using a Zeiss Axioscope2 light microscope.

### RNA extraction and semi-quantitative-RT-PCR analyses

Five uniform-size 7-days-old, *in vitro*-germinated seedlings of rice cultivar Nipponbare were transplanted to a magenta GA7 plant tissue culture box containing 25 ml of rice nutrient solution and grown for 14 days. Then, plants were transferred to rice nutrient solution with 5 μM As(III), or EA105 (OD_600_ = 0.02) or EA106 (OD_600_ = 0.02) or combinations of all. Post 24 h of treatment total RNA was isolated from pool of five whole seedlings using the Bio Basic EZ-10 Spin Column Plant RNA Mini-Prep Kit. The possible genomic DNA contaminant in RNA extract was removed using turbo DNA-free™ kit (Ambion). The quantity of total RNA was determined using NanoDrop. First-strand complementary DNAs were synthesized from 500 ng of total RNA using High Capacity cDNA Reverse Transcription Kit. PCR was carried out using standard Taq Polymerase (New England Biolabs) using the gene specific primers (Supplementary Table 1). PCR amplifications were performed using PCR mixture (15 μL) that contained 1 μL of RT reaction product as template, 1 × PCR buffer, 200 μM dNTPs (Fermentas GmbH), 1 U of Taq DNA polymerase (New England Biolab), and 0.1 μM of each primer depending on the gene (Supplementary Table 1). PCR was performed at initial denaturation at 94°C for 4 min, 24, or 26 or 28 cycles (30 s at 94°C; 30 s at 60°C; 30 s at 72°C), and final elongation (8 min at 72°C) using a Bio-rad thermal cycler (Supplementary Table 1). We performed a negative control containing RNA instead of cDNA to rule out any possible genomic DNA contamination. The PCR products were electrophoresed on 1.4% agarose gel, stained with ethidium bromide and documented in a gel documentation system; the bands were quantified using ImageJ. Each band was normalized against the intensity obtained with the same cDNA using the actin primers.

### Data analysis

Data were presented as mean with standard error. The statistical software JMP11 was used to analyses the data. The data were analyzed by one-way analysis of variance (ANOVA), and *post hoc* mean separations were performed by Tukey's HSD test and results were considered to be statistically different when *p* < 0.05.

## Results

### As treatment increases susceptibility of rice against *M. oryzae*

**Figure 1:**
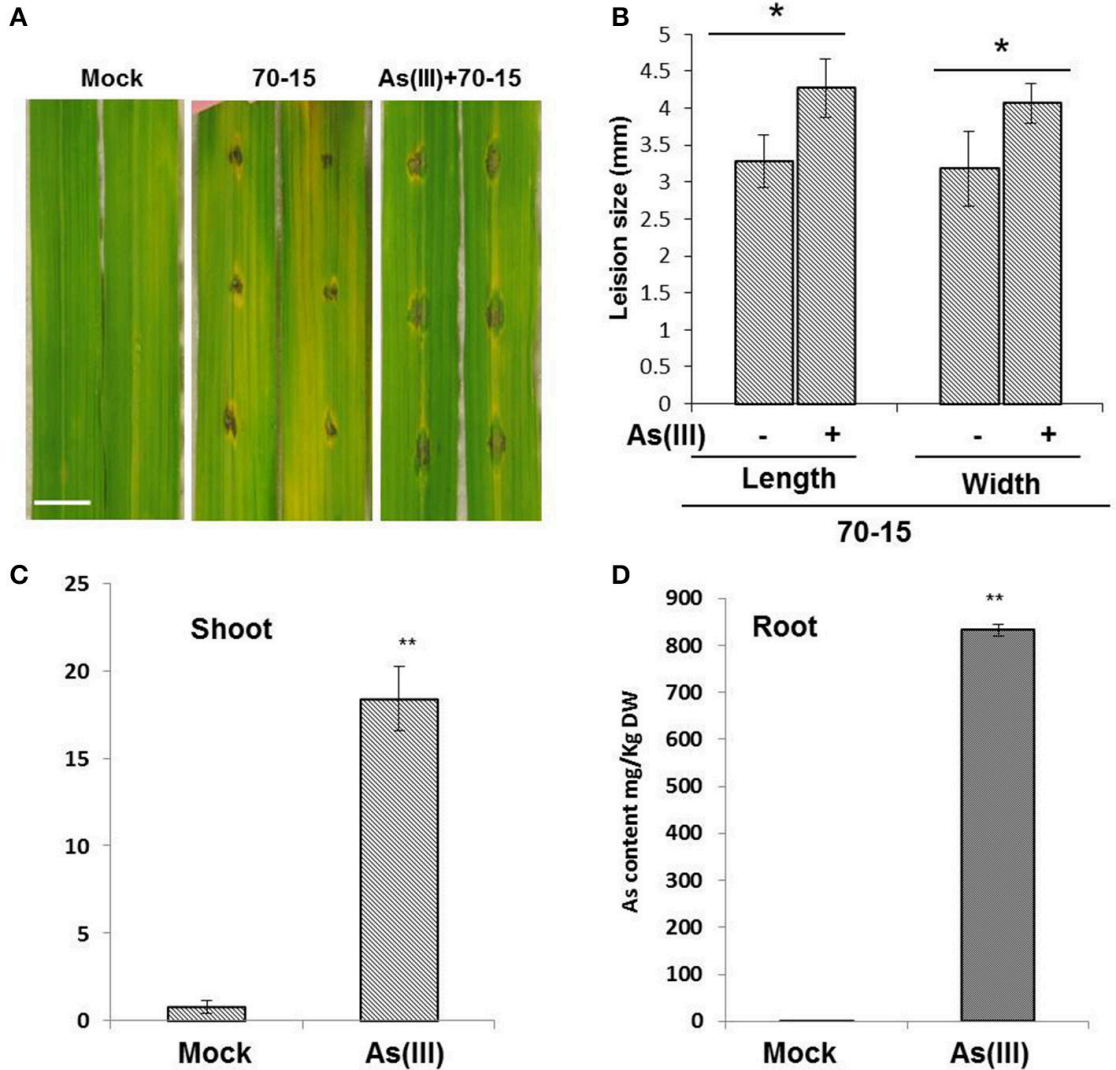
**As(III) treatment increases susceptibility of rice against ***M. oryzae*****. Rice cultivar Seraceltik was germinated on water and transferred to 8-L system containing rice nutrient solution. After 21 days the plants were treated with and without As(III) (5 μM) and incubated for 7 days. Uniform leaves were infected with *M. oryzae* 70-15 tested for their ability to form lesions. **(A)** Representative photographs were taken 5 days post-treatment (Scale bar = 10 mm). **(B)** Lesion size measured by length and width. **(C,D)** ICP-MS quantification of total As content in root and shoots of rice plants. Experiments with three replicates were repeated twice. Error bars indicate standard error. A means comparison was done within lesion length and within lesion width. *P ≤ 0.05, **P ≤ 0.01; two-tailed Student's *t*-test.

To evaluate the impact of As treatment on rice blast infections, 21 days old Seraceltik seedlings were treated with 5 μM As(III). Post 7 days treatment of 5 μM As(III), leaves of rice seedlings were subjected to *M. oryzae* infections. The *M. oryzae* were prepared following the protocol published in Spence et al. (2014a). Plant pre-treated with As(III) showed increased susceptibility against blast infections in terms of increased lesion size compared to untreated and mock plants (Figures 1A,B). Plant treated with As(III) showed significant As-uptake in aerial shoots/leaves compared to the mock treatment (Figures 1C,D). Roots showed more accumulation of total As compared to shoots, the levels of As recorded *in planta* were in accordance with the published reports (Das et al., 2016). We also tested the total As content in the rice grains post As amendment. The Nipponbare genotype was primed with EA106 and treated with As(III) for the total As grain content. Nipponbare seedlings primed with EA106 and treated with As(III) showed significant reduction in grain As content compared to untreated mock plants (Supplementary Figure 1).

### Exposure of as against *M. oryzae* and other plant pathogenic fungi

**Figure 2:**
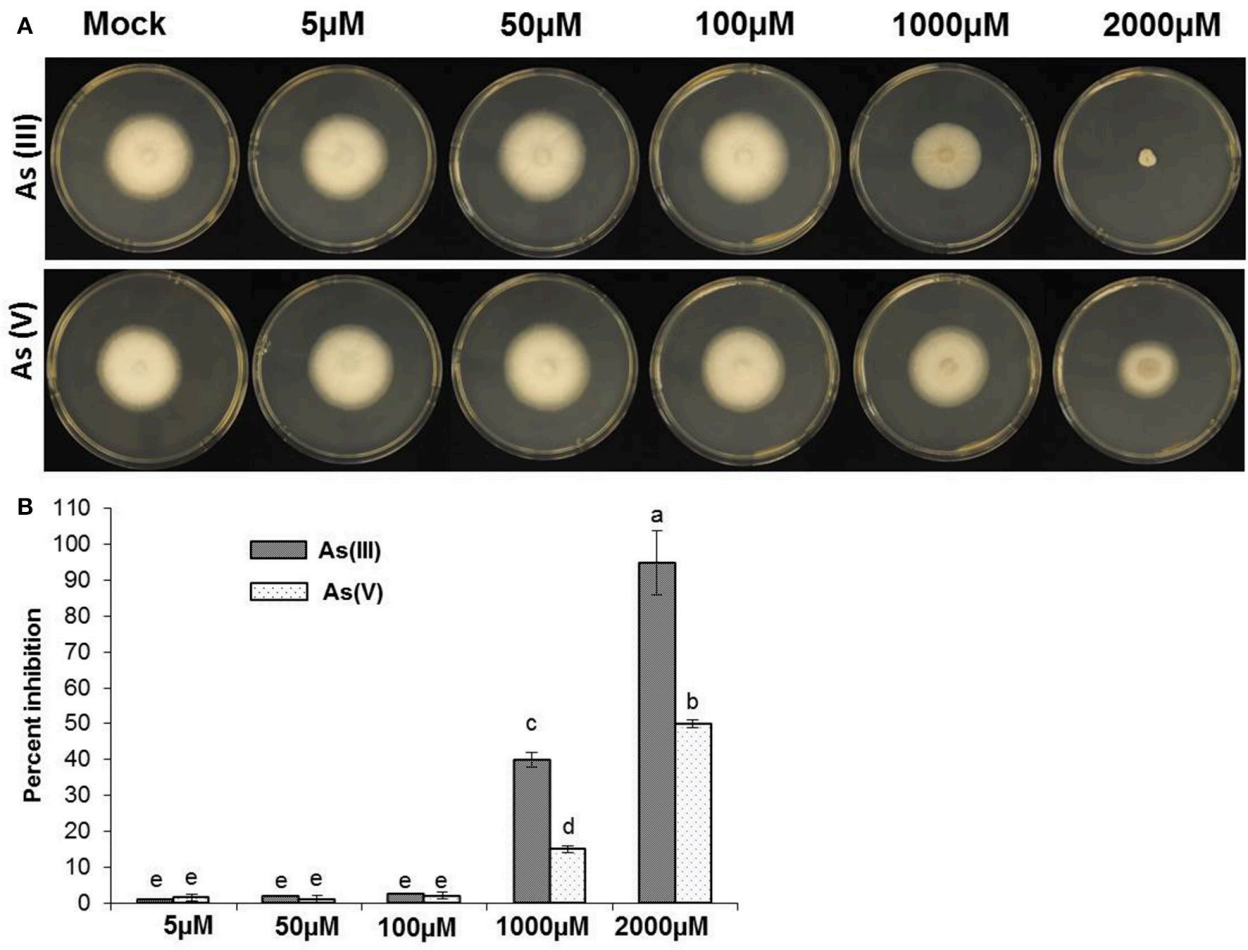
**Exposure of As against ***M. oryzae*****. 5 mm size of fungal plug from 7 days grown *M. oryzae* 70-15 was transferred to CM plates with different concentration (0, 5, 50, 100, 1000, and 2000 μM) of As(III) and As(V) and incubated for 5 days in dark. **(A)** Representative image of the fungal inhibitory effect seen when 70–15 was growth on CM plates with As(III) or As(V). **(B)** Degree of inhibition of *M. oryzae* 70–15 by adding As(III) or As(V) on CM agar plats. Each treatment had five replicates and repeated twice. Error bars indicate standard error. Different letters indicate statistically significant differences between treatments (Tukey's HSD).

Both species of As [As(III) and A(V)] are potentially toxic to both prokaryotes and eukaryotes (Styblo et al., 2000). Previously, we have observed toxic effects of As on rice plants grown hydroponically (Lakshmanan et al., 2015). To test if As inflicts toxics effects on rice blast pathogen, *M. oryzae* was exposed to increasing concentrations of both As(III) and As(V) (0–2 mM). As was supplemented in the media and a fungal plug was inoculated, radial growth post 5 days was estimated and photographed. *M. oryzae* showed significant tolerance against As(III) and As(V) at 100 μM levels (Figures 2A,B). Interestingly, *M. oryzae* showed increased tolerance against As(V) compared to As(III) at 2 mM levels (Figures 2A,B). To check the specificity of fungal tolerance against As, other non-host fungal strains were also evaluated. *Fusarium equisetti* and *Geotrichum candidum* were checked against As(III) and As(V) toxicity. In stark contrast, *F. euqisetti* showed increased susceptibility against As(III) and As(V) compared to *M. oryzae*, concentration of As over 50 μM were toxic to growth of *F. quisetti* (Supplementary Figure 2). Interestingly, *G. candidum* showed increased tolerance against both As(III) and As(V) (Supplementary Figure 2A,B).

### As treatment and appressoria formation

**Figure 3:**
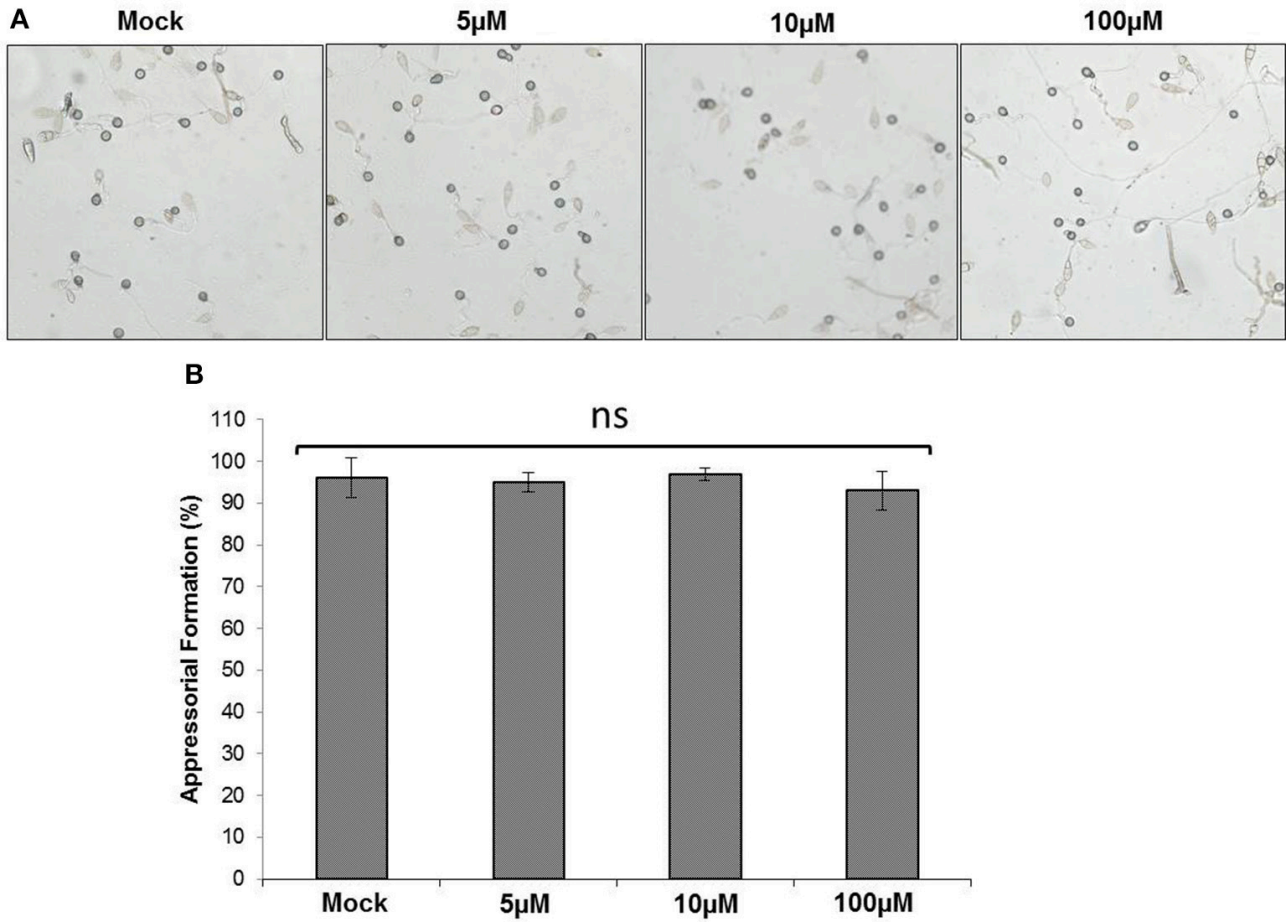
**Effect of As treatment on appressoria formation**. Spores of *M. oryzae* 70-15 were treated with different concentration (0, 5, 10, and 100 μM) As(III) and incubated for 12 h. **(A)** Representative image of appressorial formation. **(B)** Degree of appressorial formation. Each treatment had five replicates and repeated twice with similar results. Error bars indicate standard error. No statistical differences between treatments (Tukey's HSD).

Since an increased tolerance of *M. oryzae* against As was observed, spore germination and appressoria formation in *M. oryzae* post As(III) treatment was evaluated. *M. oryzae* was exposed to increasing concentration of As (0–100 μM) and spore germination and appressoria formation was evaluated per the published protocol (Spence et al., 2014a). No significant reduction in spore germination and appressoria formation was observed post As (III) treatment in *M. oryzae* (Figure 3).

### Rhizobacterial inoculation and blast infection in as treated rice plants

**Figure 4:**
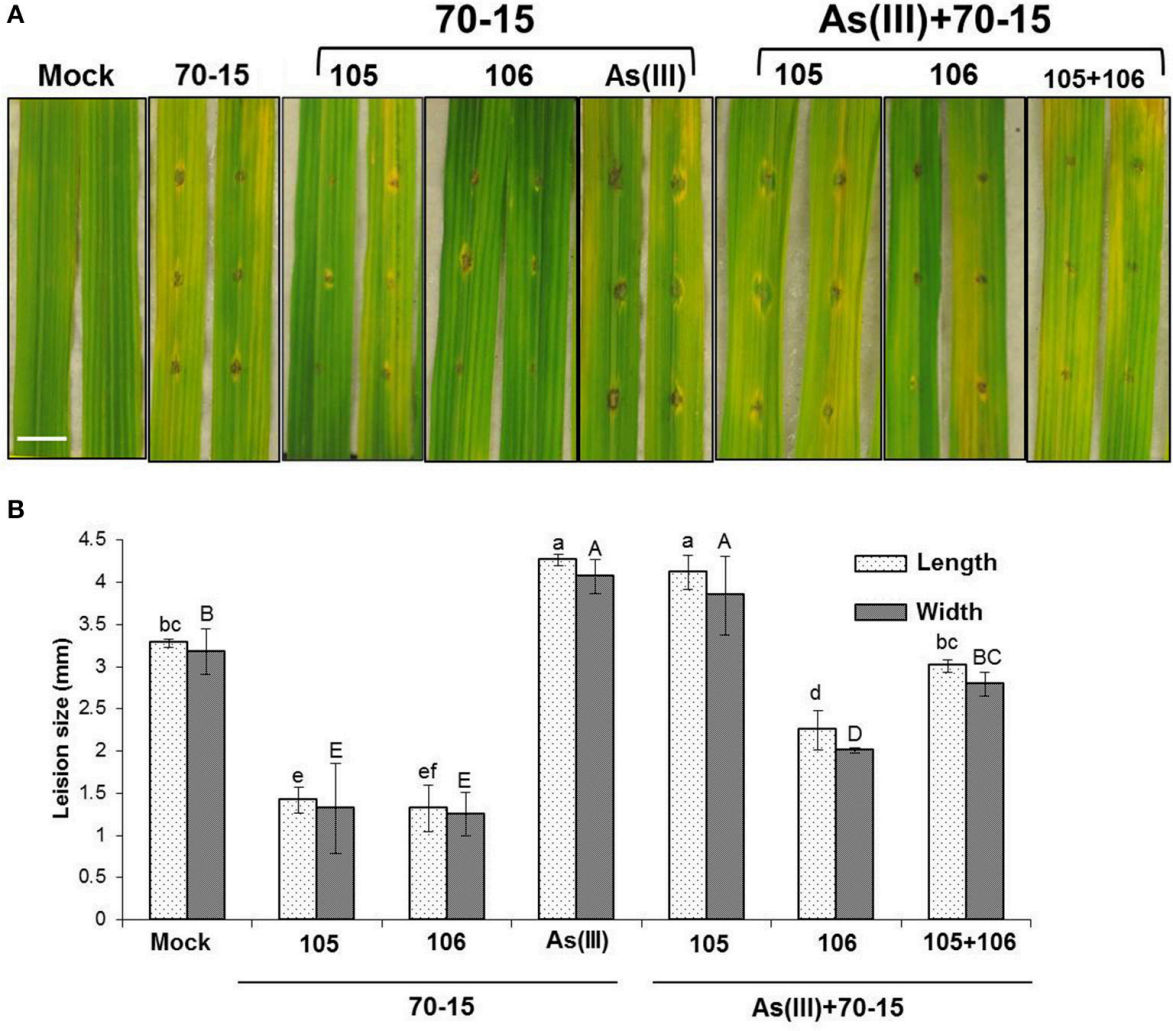
**Microbial inoculation and As treatment modulates blast infection in rice plants**. Rice cultivar Seraceltik was germinated on water and transferred to 8-L system containing rice nutrient solution. After 21 days the plants were treated with natural isolates EA105 or EA106 with or without (5 μM) As(III) and incubated for 7 days. Two leaves per plants were used for *M. oryzae* 70-15 cut leaf assay. **(A)** Representative images for *M. oryzae* 70-15 infection after 5 days post-treatment (Scale bar = 10 mm). **(B)** Degree of *M. oryzae* 70-15 infection measured by lesion size measured by length and width. Each treatment had five plates and repeated twice with similar results. Error bars indicate standard error. Different letters indicate statistically significant differences between treatments (Tukey's HSD).

Previously, it was shown that two different natural rice isolates from rice Rhizosphere impact both As uptake and blast infections in rice (Spence et al., 2014a; Lakshmanan et al., 2015). Isolates such as EA105 and EA106 were shown to reduce blast infections and As uptake respectively in rice plants. In here, it was envisaged to treat rice plants with the co-inoculation of natural isolates (EA105, EA106, and together) to evaluate plants response against mixed stress regime of As and blast infections. Three-week old rice plants were root-primed with EA105, EA106 and a mixed inoculum of EA105 + EA106 per the published protocol (Spence et al., 2014a; Lakshmanan et al., 2015). Plants were simultaneously subjected to As(III) (5 μM) treatment. Post 7 days of inoculation and As administration plants were subjected to blast infections. In accordance with the earlier observations both EA105 and EA106 reduced disease incidence and blast infection in rice compared to mock untreated plants (Figure 4). Plants primed with As in soil and co-inoculated with EA105/106 or EA105 + 106 showed increased susceptibility to blast infections compared o As-unprimed plants (Figure 4). Interestingly, EA105 and As treated plants showed more susceptibility to blast compared to mock and lone EA105 treated plants (Figure 4). Co-inoculation of EA105 and 106 in an As rich environment lead to decrease in blast incidence compared to EA105 or mock treated plants (Figure 4).

### Rhizobacterial inoculation modulates as uptake

**Figure 5:**
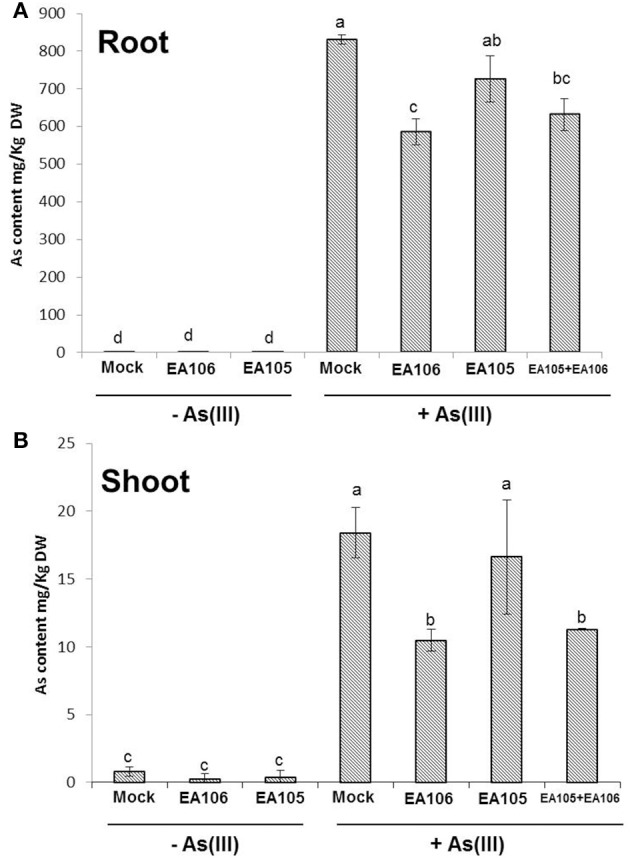
**Rhizobacterial inoculation modulates As uptake**. Rice cultivar Seraceltik were germinated on water and transferred to 8-L system containing rice nutrient solution. After 21 days the plants were treated with natural isolates EA105 or EA106 with or without (5 μM) As(III) and incubated for 7 days. ICP-MS quantification of As content in roots **(A)** and shoots **(B)** of rice plants. Each treatment had three plants and repeated twice with similar results. Error bars indicate standard error. Different letters indicate statistically significant differences between treatments (Tukey's HSD).

The results showed that plants pre-treated with As revealed increased disease incidence and progression against rice blast fungus *M. oryzae*. Interestingly, plants pretreated with As and bacterial inoculum EA106 showed reduced disease incidence. To elucidate how bacterial inoculation impacted As uptake, we used both EA105 and 106 inoculation in rice plants. Three-weeks old rice plants (Nipponbare) were supplemented with As(III) (5 μM) and were co-inoculated with EA105, EA106, and EA105+EA106 (OD_600_ = 0.02). Post inoculations plants were analyzed for total As content in roots and shoots. Expectedly, EA106 inoculation led to less uptake of As in the aerial parts of the plants compared to mock plants (Figures 5A,B). Contrastingly, EA105 treatment didn't impact the As uptake and was similar to untreated plants (Figures 5A,B). Interestingly, plants co-inoculated with EA105 and EA106 showed significant reduction in shoot As content compared to mock and EA105 treated plants (Figures 5A,B). The total root As content also showed reduction with EA106 treatment but was not significant under the EA105 and EA106 co-inoculation regime (Figures 5A,B).

### Transcriptional response of defense-related genes in as and as + microbial inoculum treated rice plants

To evaluate the effect of As treatment on rice plant defense, transcriptional response of classical defense-related genes were evaluated. Rice plants (21 days) grown under sterile conditions were subjected to As(III)(5 μM) and rhizobacterial treatments. Total RNA was isolated from rice plants subjected to As and As + rhizobacterial inoculum treatment and expression of defense related genes such as Pathogenesis-Related protein (*PR1*), Jasmonate Resistant (*JAR1*) and Ethylene Insensitive_3_-Like gene (*EIL1*) were evaluated. Expectedly, lone treatments with EA105 and EA106 induced defense response in rice plants (Supplementary Figure 3). In addition, As(III) treatment failed to induce the expression of *PR1, EIL1* and *JAR1* (Supplementary Figure 3). The co-inoculation of As with microbial inoculums (EA106 and EA106 + EA105) negated any significant changes in expression of defense-related genes (Supplementary Figure 3). Interestingly, plants treated with As and EA105 showed downregulation in PR1 expression compared to lone EA105 treatments (Supplementary Figure 3).

## Discussion

Influence of natural sediment of heavy metals including arsenic (As), cadmium (Cd), aluminum (Al) have remained as a significant environmental predicament with a negative probable impact on human health and plant productivity. Arsenic is a highly toxic heavy metal and, when present in the environment in excessive amounts, can cause serious damage to all organisms-including plants. The rhizosphere of the plant is crucial microenvironment and may have the greatest influence on the bacterial community in the soil and leading to As bioavailability and uptake into plants. We have identified a suite of nonpathogenic, rice-associated bacteria, *Pantoea sp.* (EA106) from roots of rice grown in North American rice paddy fields and have shown that they promote healthy rice growth and enhance the oxidizing potential of the rhizosphere (Lakshmanan et al., 2015). In addition, in our efforts to characterize cultivable microbiome from rice, we isolated benign microbe, *Pseudomonas sp.* (EA105) that can attenuate rice blast fungus *M. oryzae* infections (Spence et al., 2014a, 2015). The application of using benign microbes may protect plants against both biotic and abiotic stress on rice. Our previous studies showed that application of rice roots with EA106 induces iron-plaque formation abating As uptake (Lakshmanan et al., 2015). Plants are constantly challenged with both biotic and abiotic stress in nature, and the implications of combined stress regimes on plants are very poorly understood. Earlier studies involving combined stress regimes in plants showed that priming plants with one stress regime may induce resistance against a different stress response (Ben Rejeb et al., 2014). We speculated that rice plants exposed to As may show modulation in its response to rice blast infections. As expected, As pre-treated plants when challenged with *M. oryzae* infections revealed increased susceptibility against blast (Figure 1). Arsenic exposure to rice seedlings and lettuce plants modulate the structure of cellular membranes, effecting their permeability, leading to oxidative bursts, and modulates antioxidant systems and plant become weaker and more susceptible to infection (Shri et al., 2009; Tuli et al., 2010; Gusman et al., 2013). It is shown that tomato plants exposed to As modifies peroxidase responses leading to increased infections by Cucumber mosaic virus (CMV) (Miteva et al., 2005). There is evidence that plants do respond differently to a simultaneous stress compared to a standalone stress regimes. In this research, we showed that Arsenic accumulation in rice plants elevated the growth of fungus blast pathogen and the questions pertaining to how an uptake of As modifies aboveground plant response is discussed.

The invasiveness of rice blast fungi *M. oryzae* on hosts is well characterized. Spore germination, formation of appressoria as well as vegetative growth is critical for infection (Dean et al., 2012). The factors such as spore germination and appressoria formation in *M. oryzae* are also critically regulated by variety of environmental stressors including nutrients and interactions with other microbes (Mathioni et al., 2011). While almost nothing is known about *M. oryzae* interactions with heavy metals, it is tempting to speculate that the persistence of rice blast fungus may get affected by toxic elements such as As. Interestingly, rice blast fungus *M. oryzae* (up to 100 μM) and other non-host fungi *F. equisetti* (up to 50 μM) and *G. candidum* (up to 100 μM) showed tolerance when treated with As(III) and As(V) (Figure 2, Supplementary Figure 2). Our data showed that As (at near biological concentration) inflicted very limited toxicity on mycelial growth, spore germination and appresoria formation in *M. oryzae*. The effect of both biotic and abiotic components other than classical fungicides is rarely tested on the physiology and growth of rice blast fungus *M. oryzae* (Hörger et al., 2013). Previously, we reported that plant growth regulator abscisic acid (ABA) triggers *M. oryzae* spore germination and appressoria formation (Spence et al., 2015). Interestingly, *M. oryzae* biosynthesize ABA to increase its virulence in terms of appresoria formation. Lack of ABA biosynthetic genes led to decrease in appressoria formation and loss of virulence on rice plants (Spence et al., 2014a, 2015). Likewise, As and plant growth hormones like auxins, cytokinins negated any influence on spore germination and appressoria formation (Figure 3).

**Figure 6:**
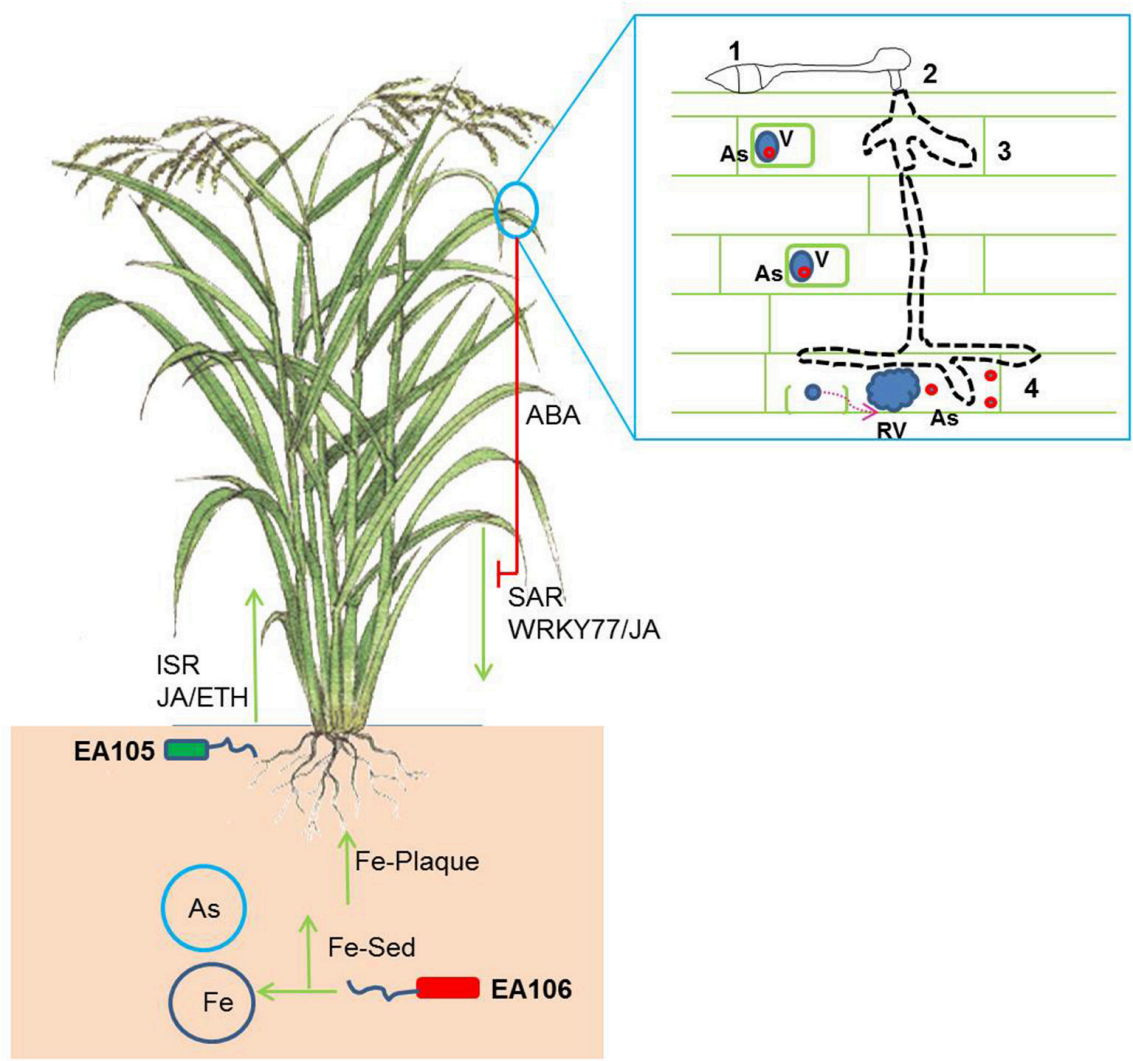
**A schematic showing the complex multi-trophic interaction in rice subjected to rhizospheric microbes, As-rich environment and blast infection**. The natural rice isolates EA105 and EA106 trigger induced systemic defense response (ISR) against rice blast infection. EA106 mobilizes rhizospheric iron (Fe) *via* siderophore (Fe-Sed) activity and triggers Fe-plaque formation on roots impeding As mobilization in plants. *M. oryzae* infection also induces systemic acquired defense (SAR) response in plants leading to resistance against blast infection. In addition, blast fungus suppresses SAR by producing abscisic acid (ABA). The implication of As (red circles) cellular localization may also be modulated under *M. oryzae* infection. As localized in specialized organelles (blue circles- v = vacuoles) may get released in planta post disruption of cellular organelles (RV = ruptured vacuoles) by rice blast. The boxed panel shows different stages of infection of rice blast: 1. Germ tube and appresorria formation; 2–3. Peg formation and mycelial growth; 4. Cellular rupture by mycelial growth and As release.

Several metal hyper-accumulating plant species such as *Noccaea caerulescens* is resistant to aboveground pests such as slugs, locusts and caterpillars(Pollard et al., 2002; Behmer et al., 2005; Jiang et al., 2005) Other Nickel (Ni) and As hyper-accumulating plants such as *Brassica juncea* and *Pteris vitatta* reveals resistance against aphids and grasshoppers (Boyd and Jhee, 2005; Rathinasabapathi et al., 2007). To add to the complexity of these unusual interactions, researchers were not able to reproduce the anti-herbivory effect in field grown plants of *N. caerulescens* (Noret et al., 2007). It is been argued that the herbivory may not be a driving force for the plants to evolve to hyper-accumulate toxic elements (Hörger et al., 2013). Interestingly, there is also evidence that herbivores may use toxic metals for their own defense (Freeman et al., 2009). In contrast to the impact of heavy metal hyper-accumulation on herbivory, there have been very few studies reporting the effect of heavy metals on pathogen defense or vice versa. Studies in Ni-hyperaccumulators such as *N. caerulescens* and *Alyssum* species have been shown to be resistant to oomycetes infections (Boyd et al., 1994; Ghaderian et al., 2000). It is argued that the metal defense against pathogens is primarily dependent on mode of infection. In hyperaccumulator plants the toxic metal is usually bound to a ligand and stored in special organelles such as vacuoles (Hörger et al., 2013). Biotrophic and hemi-biotrophic fungi and bacteria may experience apoplastic metals and necrotrophic fungi which targets and disrupts cell organelles may get exposed organelle-bound metals (Hörger et al., 2013). Non-hyperaccumulators such as rice are exposed to both As rich environment and the rice blast. As is usually stored in the aerial tissues bound to a ligand such as phytochelatins (PC). Very little is known about As cellular and sub-cellular localization in non-hyper accumulator plants. It is assumed that non-hyperaccumulators localize As-PC complexes in vacuoles (Vögeli-Lange and Wagner, 1990). Rice blast fungus *M. oryzae* acts as both a biotroph and hemi-biotroph and requires different cellular machinery to invade plants under each life stages (Koeck et al., 2011). Our results showed that *M. oryzae* to be resistant against biological concentration of As. We speculate that *M. oryzae* under both biotrophic or hemi-biotrophic life stage may get As exposure *in planta*. It also known that host vacuole maintenance under the biotrophic invasion by *M. oryzae* plays a key role in blast infections (Mochizuki et al., 2015). The disruption of cellular organelles during mycelial penetration by *M. oryzae* may release As from the vacuole, which may also be toxic to plants (Figure 6).

In recent years, microbes or microbial based products are commercially used for plant growth promotion, plant protection, and bioremediation (Reviewed by Bhattacharyya and Jha, 2012; Bashan et al., 2014; Gaur et al., 2014; Nadeem et al., 2014; Lakshmanan et al., 2015; Kumari et al., 2016; Vejan et al., 2016) More recently, it was shown that natural isolates will have more implication on plant health and fitness by compatible interaction and robust colonization with the same host compared to unrelated isolates (Chen et al., 2013; Spence et al., 2014a; Lakshmanan et al., 2015). In a similar way, we have isolated a bacterium, EA105 from rice rhizospheric soil which showed direct *in vitro* inhibition of *M. oryzae* vegetative growth as well as an ability to interfere with the formation of appressoria, a structure that is critical during *M. oryzae*'s invasion of rice (Spence et al., 2014a). In addition, our work also showed isolation of yet another rice rhizospheric isolate, and EA106 which induces Fe-plaque formation and abates As uptake in roots (Lakshmanan et al., 2015). We hypothesized that microbial inoculums that attenuates As uptake and rice blast infection in rice may modulate disease progression in rice plants exposed to mixed stress regime. As explained in the earlier sections that As treatments alleviates *M. oryzae* infections. However, when As treated plants were co-inoculated with natural rice isolates it lead to decrease in disease severity (Figure 4). Interestingly, EA105 shows direct antagonistic activity against *M. oryzae* compared to EA106 (Spence et al., 2014a). In contrast, both EA105 and EA106 induce ISR against *M. oryzae* (Spence et al., 2014a). It has been clearly shown that rice rhizospheric isolates EA105 and EA106 activates resistance by induction of defense signaling molecules such as salicylic acid (SA), jasmonic acid (JA), or ethylene (ETH) (Chisholm et al., 2006; Fu and Dong, 2013; Pieterse et al., 2014; Spence et al., 2014a). Recently showed that natural isolates of rice rhizopheric soil (Spence et al., 2014a,b, 2015) and bulk soil isolates (Shimoi et al., 2010) induces JA/ET/SA related genes and attenuates *M. oryzae* infections. The specificity of rhizobacterial treatment of EA105 and EA106 to elicit PR related genes expression, thereby enhancing plant fitness, suggests that plants acts and responds differently when exposed to multiple stressors. Our data clearly showed that a co-inoculation of two different rice rhziospheric isolates could induce defense against multiple stressors (As and rice blast) in rice. The induction of classical PR defense genes post EA105/EA106 treatment may contribute toward resistance against blast infections. We have shown previously that one of the natural rice isolate EA106 abates As uptake in rice as it induces Fe-plaque formation in roots which may impede As uptake (Lakshmanan et al., 2015). Surprisingly, EA106 also induced PR genes as EA105 which would have contribute to defense against blast. Interestingly, EA105 which protects rice against blast infections didn't reduce As uptake. Surprisingly, co-treatment of As with EA105 reduced the intensity of PR1 expression compared to EA105 lone treatments. At this juncture, we don't know if As toxicity could be linked to pathogen defense in rice. These two responses may very well be compartmentalized in plants and may only have downstream impact on each other. The ability of rice blast fungus to switch from biotroph to a hemi-biotroph mode may lead to As release from the cellular organelles to cytoplasm leading to accelerate cell death and reduction in plant growth (Figure 6). Induction of PR genes and defense response post microbial inoculum treatment may play a role in delaying pathogen ingression and release of As in the apoplast.

In this study, we showed for the first time that As accumulation in rice plant tissue leads to increased susceptibility to blast fungus *M. oryzae.* The pre-treatment of rice isolates EA105 and EA106 significantly increased plant fitness by arresting the growth of blast fungus *M. oryzae*. Moreover, As uptake from root to shoot and As accumulation in shoot and grain decreased significantly as a result of the rhizobacterial inoculation. This study further strengthens the case for the use of natural isolates as a biologicals to mitigate biotic and abiotic stress response in plants. The identified natural isolates of rice microbiome can be employed for further characterization of key genes pertaining to defense responses when grown in As contaminated soil. Further research mainly focused on transcriptional and biochemical changes in rice plants growing As-contaminated soil exposed to *M. oryzae*, and co-inoculation of EA105 and EA106 provides insights into use of mixed beneficial microbes. The use of plant growth-promoting rhizobacteria such as EA105 and EA106 offer promise to be a better alternative as biocontrol agent for potential application in sustainable rice production of As-contaminated and *M. oryzae* predominant rice cultivated areas.

## Author contributions

HPB conceived the research. VL and JC conducted the experiments and drafted the manuscript. HPB provided suggestions and improvements on the manuscript. All authors read and approved the manuscript.

### Conflict of interest statement

The authors declare that the research was conducted in the absence of any commercial or financial relationships that could be construed as a potential conflict of interest.
