# Supplementary material for: Killing Two Birds with One Stone: Natural Rice Rhizospheric Microbes Reduce Arsenic Uptake and Blast Infections in Rice
Source: Front Plant Sci. 2016 Oct 13;7:1514. doi: 10.3389/fpls.2016.01514 (PMC5062539; doi:10.3389/fpls.2016.01514)
Supplement: Supplementary file 1 [file Presentation1.PDF]

# Supplementary Figure 1.

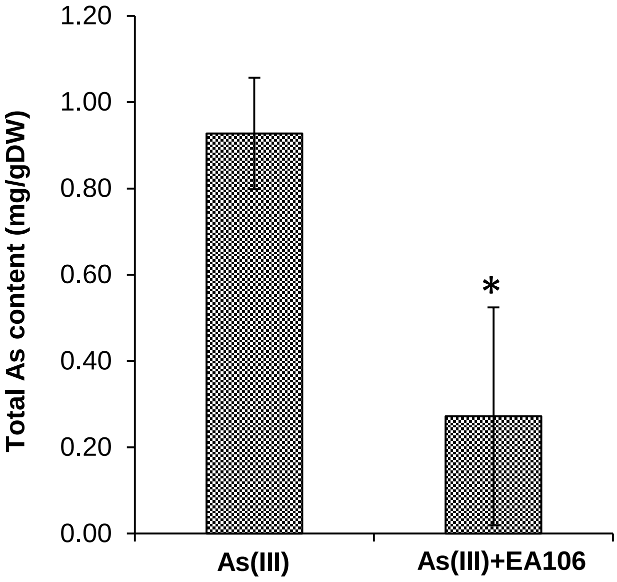


**SOM Figure 1:** ICP-MS quantification of As content in seeds of rice cv. Nipponbare . Error bars indicate standard error. *P ≤ 0.05, two-tailed Student’s t test.

# Supplementary Figure 2.

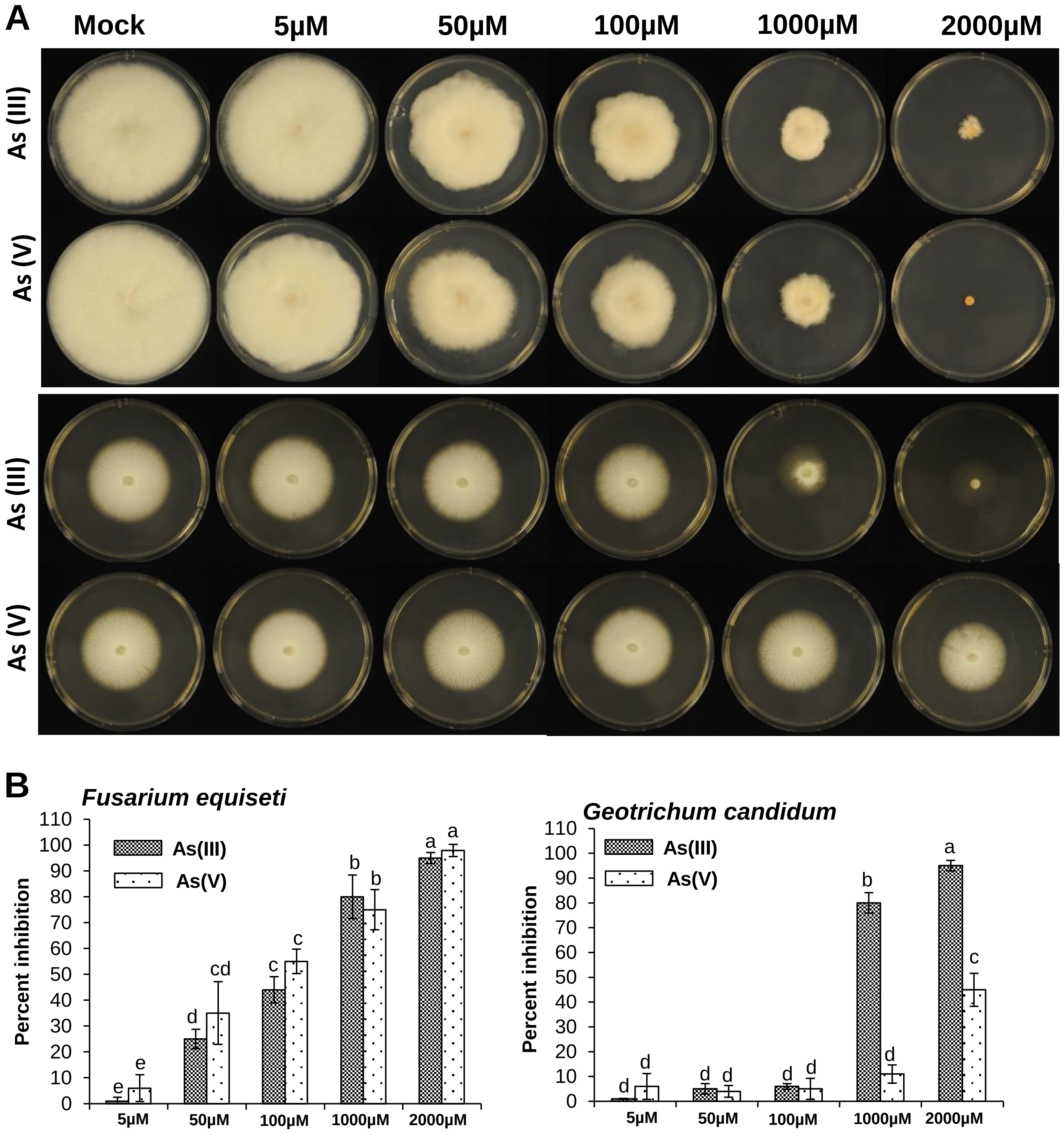


**SOM Figure 2. Exposure of As against various plant pathogenic fungi.** 5mm size of fungal plug from 7d grown *Fusarium equiseti* and *Geotrichum candidum* was transferred to PDA plates with different concentration (0, 5, 50, 100, 1000 and 2000μM) of As(III) and As(V) and incubated for 5d in dark. A) Representative image of the fungal inhibitory effect with As(III) or As(V). B) Degree of inhibition of fungal by adding As(III) or As(V) on PDA plats. Each treatment had five plants and repeated three times with similar results. Error bars indicate standard error. Different letters indicate statistically significant differences between treatments (Tukey's HSD).

# Supplementary Figure 3.

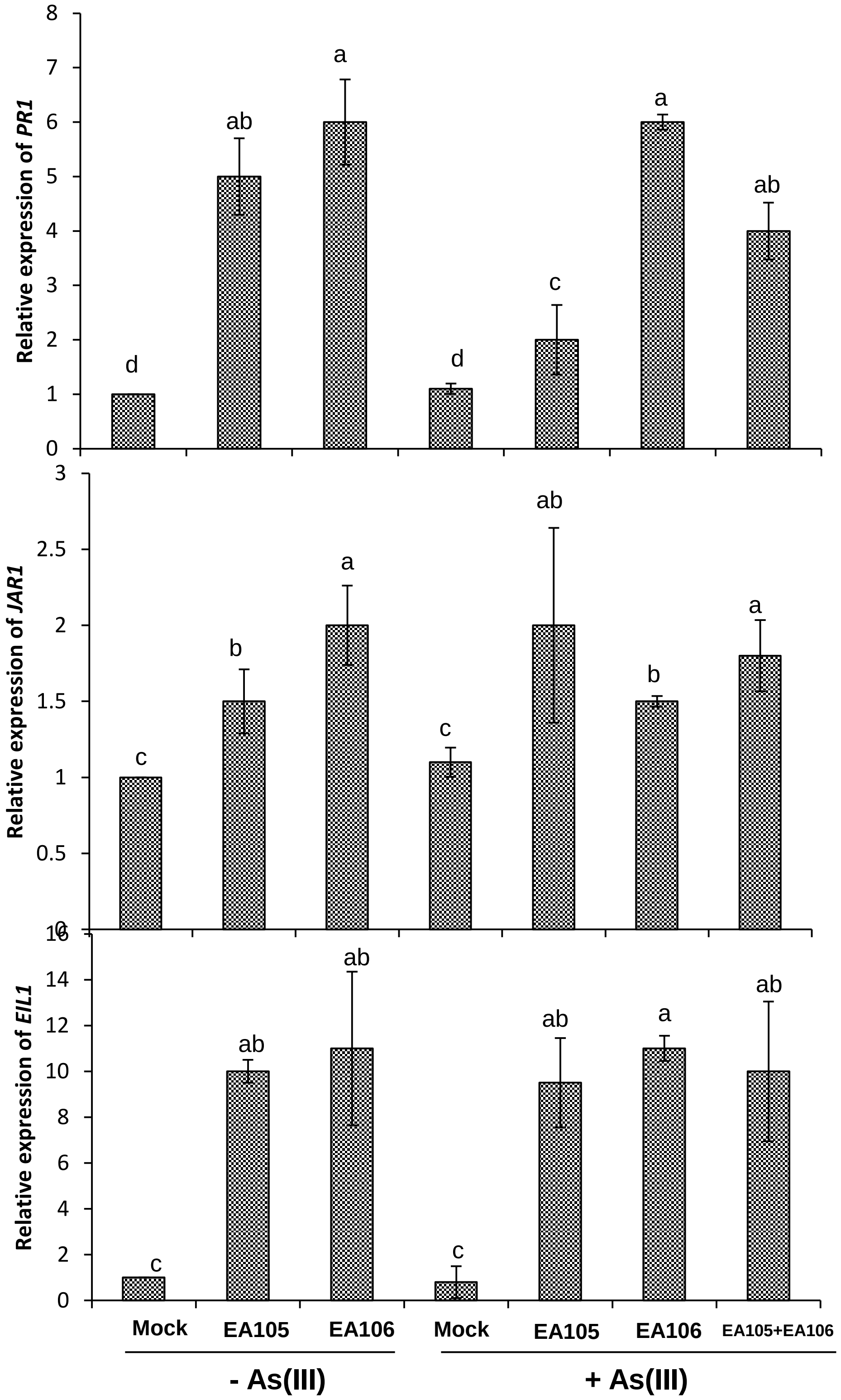


**Supplementary figure 3.** Measurement of relative expression of various defense related genes *PR1*, *JAR1*, and *EIL1* and of rice by semi-quantitative RT-PCR. Rice cultivar Nipponbare seeds were germinated on water, then transferred to magenta box containing rice nutrient solution for 7 d. Then transferred to rice nutrient solution with rhizobacteria EA105 or EA106 with or without As(III) (5 µM) or combination of all and complete rice nutrient solution as the experimental control. Total RNA was isolated after 24 h. Each treatment had three replicates. Data represent mean ± SE. Error bars indicate standard error. Different letters indicate statistically significant differences between treatments (Tukey's HSD).

**Supplementary Table 1.** Specific primers, annealing temperatures and total numbers of amplification cycles used for semi-quantitative RT-PCR.

| Primer | Primer sequence (5'-3') | Annealing temperature (ºC) | Total number of amplification cycles |
|---|---|---|---|
| ***PR1* forward** | TCGTATGCTATGCTACGTGTTT | 62 | 26 |
| ***PR1* reverse** | CACTAAGCAAATACGGCTGACA | | |
| ***EIL1* forward** | ATCACCAGCGCCATATCGTT | 60 | 28 |
| ***EIL1* reverse** | CACGGTTGTTCAGCATCAGC | | |
| ***JAR1* forward** | TCTCCCCAGCCTTAACCGTA | 60 | 28 |
| ***JAR1* reverse** | CTAAACGCGACGACAAACCC | | |
| ***Actin* forward** | GGCATCACACCTTCTACAAC | 55 | 24 |
| ***Actin* reverse** | ATCACCAGAGTCCAACACAA | | |
